# Small single perivascular hepatocellular carcinoma: comparisons of radiofrequency ablation and microwave ablation by using propensity score analysis

**DOI:** 10.1007/s00330-020-07571-5

**Published:** 2021-01-05

**Authors:** Chao An, Wang-Zhong Li, Zhi-Mei Huang, Xiao-Ling Yu, Yu-Zhi Han, Fang-Yi Liu, Song-Song Wu, Jie Yu, Ping Liang, Jinhua Huang

**Affiliations:** 1 Department of Minimal Invasive Intervention, State Key Laboratory of Oncology in South China, Collaborative Innovation Center for Cancer Medicine, Sun Yat-sen University Cancer Center, Guangzhou, China; 2 Department of Nasopharyngeal Carcinoma, Sun Yat-Sen University Cancer Center, Guangzhou, 510060 China; 3 Department of Interventional Ultrasound, State Key Laboratory of Kidney Disease, The Chinese PLA General Hospital, Beijing, 100853 China; 4 Shengli Clinical Medical College of Fujian Medical University, Fuzhou, 350001 China

**Keywords:** Hepatocellular carcinoma, Liver neoplasms, Radiofrequency ablation, Microwave, Ablation techniques

## Abstract

**Objectives:**

We aimed to compare the therapeutic outcomes of radiofrequency ablation (RFA) and microwave ablation (MWA) as first-line therapies in patients with small single perivascular hepatocellular carcinoma (HCC).

**Methods:**

A total of 144 eligible patients with small (≤ 3 cm) single perivascular (proximity to hepatic and portal veins) HCC who underwent RFA (*N* = 70) or MWA (*N* = 74) as first-line treatment were included. The overall survival (OS), disease-free survival (DFS), and local tumor progression (LTP) rates between the two ablation modalities were compared. The inverse probability of treatment weighting (IPTW) method was used to reduce selection bias. Subgroup analysis was performed according to the type of hepatic vessels.

**Results:**

After a median follow-up time of 38.2 months, there were no significant differences in OS (5-year OS: RFA 77.7% vs. MWA 74.6%; *p* = 0.600) and DFS (5-year DFS: RFA 24.7% vs. MWA 40.4%; *p* = 0.570). However, a significantly higher LTP rate was observed in the RFA group than the MWA group (5-year LTP: RFA 24.3% vs. MWA 8.4%; *p* = 0.030). IPTW-adjusted analyses revealed similar results. The treatment modality (RFA vs. MWA: HR 7.861, 95% CI 1.642–37.635, *p* = 0.010) was an independent prognostic factor for LTP. We observed a significant interaction effect of ablation modality and type of peritumoral vessel on LTP (*p* = 0.034). For patients with periportal HCC, the LTP rate was significantly higher in the RFA group than in the MWA group (*p* = 0.045). However, this difference was not observed in patients with perivenous HCC (*p* = 0.116).

**Conclusions:**

In patients with a small single periportal HCC, MWA exhibited better tumor control than RFA.

**Key Points:**

*• Microwave ablation exhibited better local tumor control than radiofrequency ablation for small single periportal hepatocellular carcinoma.*

*• There was a significant interaction between the treatment effect of ablation modality and type of peritumoral vessel on local tumor progression.*

*• The type of peritumoral vessel is vital in choosing ablation modalities for hepatocellular carcinoma.*

**Supplementary Information:**

The online version contains supplementary material available at 10.1007/s00330-020-07571-5.

## Introduction

Surgical resection (SR), liver transplantation, and local-region ablation have been recommended as first-line therapeutic options for patients with early-stage hepatocellular carcinoma (HCC) [1, 2]. Of the various ablation modalities, radiofrequency ablation (RFA) and microwave ablation (MWA) are frequently used as alternative therapeutic options for unresectable HCC in China [3–5]. In clinical practice, perivascular HCC was defined as a tumor nodule abutting the first- or second-grade branches of major vessels, including the hepatic and portal veins, with an axial diameter larger than 3 mm and the shortest distance of less than 5 mm [6, 7]. While several studies have reported on the comparative survival benefits between RFA and MWA during HCC treatment for patients fulfilling the Milan standard [6–8], few studies have compared RFA and MWA therapeutic outcomes for small (≤ 3 cm) single perivascular HCC.

Topographical factors can impede the therapeutic effectiveness of ablation therapy [9–11]. These factors include nodule-abutting organs such as the gastrointestinal tract, diaphragm, or major vessels. These organs are regarded as “challenging locations.” Technically, MWA relies on rapid heating and friction between molecules in the tumor, which is superior to RFA owing to the higher intratumoral temperature; the shorter operation time duration, the more massive cell necrosis. It is less susceptible to variations in the morphology of the ablative area resulting from heat sink effects from the adjacent vessels [3, 12, 13]. Compared with MWA, RFA treatment in perivascular HCC may result in a cold zone easily due to the slow warming of the target area, and heat dissipation result from rapid blood flow. Therefore, the local tumor control exhibited by RFA in perivascular HCC patients has not been well elucidated [14].

There is limited data on comparisons between RFA and MWA as first-line therapeutic options in patients with small single perivascular HCC. In this study, we compared the effectiveness and survival outcomes of these two ablation therapies. Given the potential selection bias in treatment assignment, we adopted the inverse probability of treatment weighting (IPTW) based on the propensity to receive treatment to reduce these bias [15].

## Materials and methods

### Study participants

As a retrospective multicenter study, ethical approvals were obtained from the Research Ethics Committees of (Sun Yat-sen University Cancer Center, the Chinese PLA General Hospital, and the Fujian Provincial Hospital). A total of 1,783 treatment-naïve patients with biopsy-confirmed HCC which fulfilled the Milan criteria and had received RFA or MWA as a first-line treatment between October 2012 and December 2018 were reviewed. The inclusion criteria were as follows: (i) a performance status of 0 or 1; (ii) presenting with a small single perivascular tumor (tumor size ≤ 3 cm; perivascular peritumoral vessel diameter > 3 mm); and (iii) availability of medical records and imaging data. The exclusion criteria included the following: (i) patients who had undergone other treatments before ablation therapy; (ii) the presence of vascular invasion or extrahepatic metastases; (iii) severe coagulopathy; and (iv) inability to follow up. The reasons for conducting thermal ablation rather than SR in these patients were as follows: (i) insufficient liver remnants; (ii) psychological resistance to invasive treatment; (iii) refusal of general anesthesia; and (iv) high risk for complications of resection associated with tumor location or old age.

### Classification of perivascular HCC

In this study, perivascular HCC was defined as a tumor nodule abutting the first- or second-grade branches of major vessels, including the hepatic and portal veins, with an axial diameter larger than 3 mm and the shortest distance of less than 5 mm [6, 7]. Perivascular HCCs were reclassified by two independent investigators (L.Z.L. and J.Z.). The baseline computer tomography and magnetic resonance imaging data were reviewed. To understand the spatial relationships between tumors and major vessels, a 3D visualization ablation planning system was used to segment the target area and generate a 3D image (Supplementary Figure 1). If the target tumor abutted more than one vessel, the largest vessel was selected for our study. Discordances between the two investigators regarding classifications were solved by inter-observer agreement analysis.

### Study variables

Demographic and clinicopathologic characteristics, including age, sex, comorbidities, etiology, cirrhosis, albumin-bilirubin (ALBI) grade, tumor size, hepatic segments, type of abutting vessels, and results of routine laboratory test, were obtained from medical records in the institutional database. The disease markers analyzed in this study included alpha-fetoprotein (AFP), aspartate aminotransferase (AST), alanine aminotransferase (ALT), albumin (ALB), and total bilirubin (TBIL). Due to its advantages, the ALBI grade, not the Child-Pugh class, was the most important criterion for evaluating liver functions in this study [16, 17]. The ALBI grade was calculated before ablation therapy using the TBIL and ALB as follows: (log TBIL [μmol/L] × 0.66) + (ALB [g/L] × − 0.085). The ALBI grade was classified as follows: grade 1 (≤ − 2.6), grade 2 (− 2.6 to − 1.39), and grade 3 (> − 1.39), respectively.

### Follow-up protocol

The success of the technique was determined by the disappearance of contrast enhancement within or abutting the ablation zone on imaging examination one month after therapy [18]. Incomplete ablation after the second ablation treatment session was defined as a technical failure. Patients with incomplete ablation who underwent a repeat ablation treatment for tumor control were excluded from the outcome analysis. Routine contrast-enhanced images and serum tumor markers were assessed at 1 and 3 months post-ablation therapy, with every 6 months follow-up after that. In cases with suspected distant metastasis, chest CT, whole-body bone scans, or positron emission tomography (PET)-CT were performed selectively. The follow-up medical records of RFA and MWA for perivascular tumors are shown in Supplementary Figures 2 and 3. In cases where tumor progression was detected during follow-up, individualized salvage therapy was performed based on the characteristics of the recurrent tumor, liver function levels, and the patient’s general condition. The optimal ablative modalities for tumor progression were determined by a multidisciplinary team.

### Study outcomes

The resulting ablation parameters and survival outcomes between the two ablation groups were compared. The primary endpoints were overall survival (OS), disease-free survival (DFS), and local tumor progression (LTP). OS was calculated from the date of initial treatment to the date of death due to any cause. Patients were censored at the last follow-up date (March 31, 2019) or the date lost to follow-up. DFS was measured from the initial treatment until tumor progression or death. LTP was defined as developing new tumor mass in the liver either around or within the ablation bed. Major complications were defined as events that caused substantive morbidities and disabilities that increased care levels or led to hospital admission, or substantially prolonged the hospital stay.

### Statistical analysis

Continuous variables were presented as mean and standard deviation (SD), while classified variables were expressed in frequencies and percentages. Continuous variables that fulfilled the normality assumption were compared using Student’s *t* test; otherwise, the Wilcoxon rank-sum test was used. Classified variables were analyzed using the chi-square test or Fisher’s exact tests, where appropriate. Inter-observer concordance on the classification of perivascular HCC was evaluated by Cohen’s kappa statistics. Survival curves were estimated using the Kaplan-Meier analysis with the log-rank test. Univariate and multivariate step Cox regression models were performed to identify prognostic factors associated with different endpoints. To explore the potential time-varying effect on treatment modalities, we used flexible parametric models provided by R package ‘rstpm2’ to model the baseline hazard flexibly. Given the potential imbalances in treatment assignment, we performed the inverse probability treatment weighting (IPTW) method to reduce observed biases between groups. Treatment propensity was calculated by logistic regression using a panel of potential confounding factors that could have affected the original therapeutic decisions. The standard mean difference (SMD) was used to evaluate the covariate balance. An SMD of less than 0.1 was considered a sign of sufficient balance. All statistical analyses were performed using R 3.6.3 and the SPSS 21.0. Statistical significance was set at *p* ≤ 0.05.

## Results

### Baseline characteristics

**Fig. 1:**
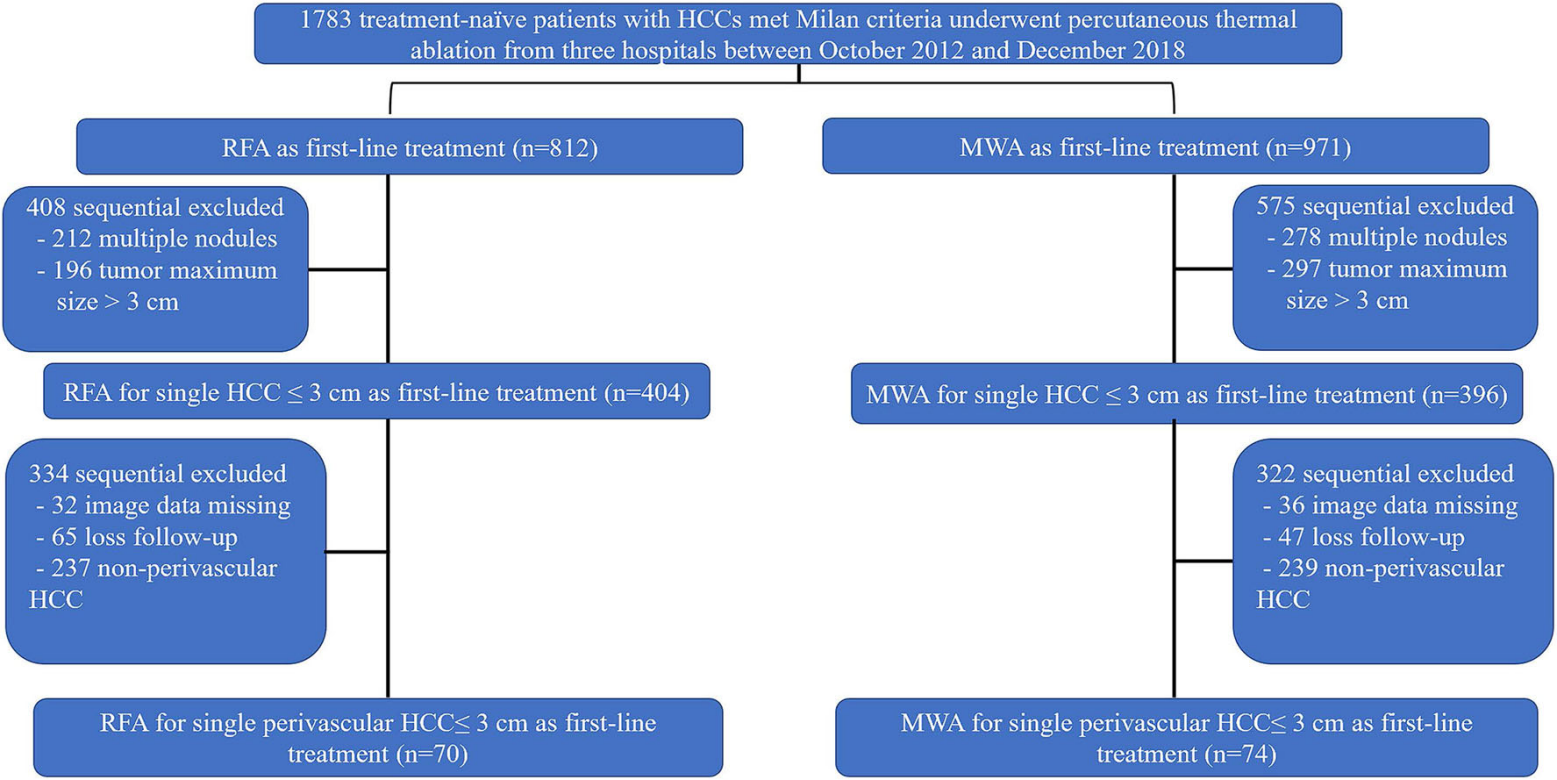
Flowchart for patient selection

Figure 1 shows the baseline characteristics of the enrolled study participants. A total of 144 patients with small single perivascular HCC treated with RFA (*n* = 70) or MWA (*n* = 74) as first-line therapy were enrolled in the study. The median follow-up for the study population was 38.2 months (range, 3.2–83.9 months). The median follow-up for the RFA and MWA groups was 38.9 months (range, 3.4–83.9 months) and 37.6 months (range, 3.2–79.2 months), respectively. Patient characteristics in unweighted and weighted cohorts stratified by ablation technique are outlined in Table 1. Standardized mean differences in the unweighted cohort showed that significant differences were observed in cirrhosis, tumor size, alanine aminotransferase, aspartate aminotransferase, total bilirubin, and albumin. To distinguish the vessel types, the examination of all the images by two radiologists revealed a final kappa value of 0.82 (95% CI: 0.72–0.96).

**Table 1:** Baseline characteristics of the study cohort stratified by treatment modality

Variables	Overall (*n* = 144)	Unweighted			Weighted		
		RFA (*n* = 70)	MWA (*n* = 74)	SMD	RFA	MWA	SMD
Age (year), mean ± SD	56.9 ± 10.8	57.4 ± 10.1	56.4 ± 11.5	− 9.5	57.0 ± 10.0	56.9 ± 11.4	− 1.1
Gender				− 9.0			− 1.9
Female	19 (13.2)	6 (8.6)	13 (17.6)		10.0	11.9	
Male	125 (86.8)	64 (91.4)	61 (82.4)		90.0	88.1	
Comorbidity				7.3			− 1.5
Absence	81 (56.2)	42 (60.0)	39 (52.7)		61.3	62.8	
Presence	63 (43.8)	28 (40.0)	35 (47.3)		38.7	37.2	
Etiology							
HBV	111 (77.1)	54 (77.1)	57 (77.0)	− 0.1	79.7	74.1	− 5.6
HCV	13 (9.0)	4 (5.7)	9 (12.2)	6.5	7.1	8.8	1.8
Other	20 (13.9)	12 (17.1)	8 (10.8)	− 6.3	13.3	17.1	3.8
Cirrhosis				10.7			-3.3
Absence	25 (17.4)	16 (22.9)	9 (12.2)		17.0	20.3	
Presence	119 (82.6)	54 (77.1)	65 (87.8)		83.0	79.7	
Child-Turcotte-Pugh grade				− 2.9			− 1.4
A	142 (98.6)	68 (97.1)	74 (100.0)		98.4	100.0	
B	2 (1.4)	2 (2.9)	0 (0.0)		1.4	0.0	
ALBI grade				2.9			− 5.9
1	76 (52.8)	38 (54.3)	38 (51.4)		55.0	60.9	
2–3	68 (47.2)	32 (45.7)	36 (48.6)		45.0	39.1	
Tumor size (cm)	2.1 ± 0.6	2.1 ± 0.6	2.0 ± 0.5	− 16.8	2.05 ± 0.6	2.05 ± 0.5	0.7
Tumor location							
I	3 (2.1)	0 (0.0)	3 (4.1)	4.1	0.0	2.1	2.1
II	9 (6.2)	2 (2.9)	7 (9.5)	6.6	3.3	5.9	2.6
III	3 (2.1)	1 (1.4)	2 (2.7)	1.3	1.5	1.9	0.4
IV	14 (9.7)	7 (10.0)	7 (9.5)	− 0.5	10.6	16.0	5.4
V	19 (13.2)	10 (14.3)	9 (12.2)	− 2.1	16.4	11.9	− 4.5
VI	24 (16.7)	13 (18.6)	11 (14.9)	− 3.7	16.4	15.2	− 1.2
VII	30 (20.8)	15 (21.4)	15 (20.3)	− 1.2	21.0	20.4	− 0.6
VII	42 (29.2)	22 (31.4)	20 (27.0)	− 4.4	30.8	26.6	− 4.2
Peritumoral vessel				− 5.3			3.0
Portal vein	80 (55.6)	37 (52.9)	43 (58.1)		48.2	51.2	
Hepatic vein	64 (44.4)	33 (47.1)	31 (41.9)		51.8	48.8	
AFP (ng/ml)	334.7 ± 1179.1	276.2 ± 770.8	390.0 ± 1468.2	− 9.7	511.3 ± 1189.5	397.6 ± 1352.8	− 9.1
ALT (U/L)	42.0 ± 43.6	50.23 ± 56.5	34.3 ± 23.9	− 36.8	42.3 ± 44.8	38.8 ± 25.8	− 8.3
AST (U/L)	42.3 ± 44.6	51.73 ± 59.2	33.4 ± 20.5	− 41.5	41.7 ± 45.1	38.2 ± 22.7	− 8.1
TBIL (μmol/L)	15.1 ± 8.43	14.2 ± 8.6	15.9 ± 8.3	19.4	15.6 ± 8.2	14.9 ± 7.2	− 9.6
ALB (g/L)	38.7 ± 8.6	37.9 ± 11.7	39.5 ± 3.9	18.2	40.3 ± 12.8	40.1 ± 3.9	− 2.2
Image-guided method				3.9			3.1
CT	65 (45.1)	33 (47.1)	32 (43.2)		48.0	44.6	
US	79 (54.9)	37 (52.9)	42 (56.8)		52.0	55.4	
Ablation sessions				2.7			2.1
1	140 (97.2)	69 (98.6)	71 (95.9)		98.6	96.5	
2	4 (2.8)	1 (1.4)	3 (4.1)		1.4	3.5	

Unless otherwise noted, continuous variables are given as mean ± standard deviation (SD), and categorical variables are given as No. (%) in overall and unweighted cohort whereas they are given as % in weighted cohort. *RFA*, radiofrequency ablation; *MWA*, microwave ablation; *SMD*, standardized mean difference; *HBV*, hepatitis B virus; *AFP*, α-fetoprotein; *ALT*, alanine aminotransferase; *AST*, aspartate aminotransferase; *TBIL*, total bilirubin; *ALB*, albumin

### Comparison of intra- and postoperative parameters

Table 2 shows the intra- and postoperative parameters. The statistical difference regarding the success rates of the techniques between the two treatment groups was not significant (RFA: 97.1% vs. MWA: 100%, *p* = 0.235). Ablative duration and power in the RFA group were significantly high compared to those in the MWA group (both *p* < 0.001). The differences in the postoperative hospitalization, cost, and major complications between the two groups were not significant.

**Table 2:** Intra- and post-ablation parameters of patients undergoing MWA and RFA

Parameter	RFA (*N* = 70)	MWA (*N* = 74)	*p* value
Procedure duration (min) (mean ± SD)	10.8 ± 2.7	6.2 ± 3.2	< 0.001
Ablative power (W)	132.8 ± 14.8	55.2 ± 5.9	< 0.001
Postoperative hospitalization (day) (mean ± SD)	4.4 ± 1.1	4.3 ± 1.5	0.598
Cost (yuan) (mean ± SD)	34843.6 ± 4352.5	32827.8 ± 3673.8	0.245
Major complication	1/70 (1.4)	1/74 (1.4)	0.968
Technique effectiveness	68/70 (97.1)	74/74 (100)	0.235

Unless otherwise indicated, numbers in parentheses are percentages. *RFA*, radiofrequency ablation; *MWA*, microwave ablation; *SD*, standard deviation; *LTP*, local tumor progression

### Comparisons of oncological outcomes before and after IPTW

**Fig. 2:**
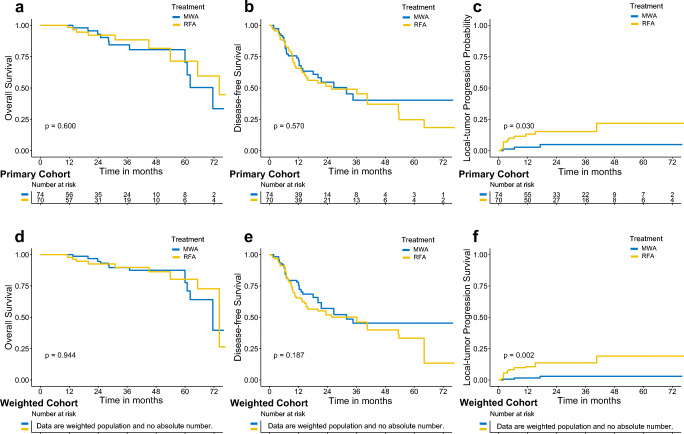
Survival curves with log-rank test stratified by treatment modalities with or without the inverse probability of treatment weighting (IPTW) adjustment. **a** Crude overall survival (OS). **b** Crude disease-free survival (DFS). **c** Crude local tumor progression probability (LTP). **d** IPTW-adjusted OS. **e** IPTW-adjusted DFS. **f** IPTW-adjusted LTP

As of March 31, 2019, 9 and 11 death events had been documented in the RFA and MWA treatment groups, respectively, while 29 and 37 recurrence events had been documented in the two groups. For recurring HCC, 86.2% and 88.2% of the patients in the RFA and MWA treatment groups were administered the same repeat ablation modality. There were no significant differences between the two groups (*p* = 0.412). In the crude Kaplan-Meier analyses, no significant differences were observed with regard to OS (cumulative 5-year OS rates: RFA 77.7% vs MWA 74.6%; *p* = 0.600; Fig. 2a) and DFS (cumulative 5-year DFS rates: RFA 24.7% vs MWA 40.4%; *p* = 0.570; Fig. 2b). A higher LTP rate was observed in the RFA group (cumulative 5-year LTP rates: RFA 24.3% vs. MWA 8.4%; *p* = 0.030; Fig. 2c) compared to the MWA group. The baseline characteristics between the two groups were balanced (all SMD < 0.1, Table 1). IPTW-adjusted Kaplan-Meier analyses also showed that, except for LTP (*p* = 0.002, Fig. 2f), there were no significant differences in OS (*p* = 0.944, Fig. 2d) and DFS (*p* = 0.187, Fig. 2e) between the two groups.

### Analysis of risk factors for OS and DFS

The results of univariate and multivariate Cox regression analyses for OS and DFS are summarized in Table 3. It is shown that OS was significantly affected by AFP levels (HR: 1.697; 95% CI: 1.226–5.461; *p* = 0.019) and ALBI grade (HR: 2.071; 95% CI: 1.891–5.410; *p* = 0.020). Tumor size (HR: 1.794; 95% CI: 1.015–3.169; *p* = 0.044) was the only independent risk factor for DFS.

**Table 3:** Prognostic factor analysis for overall survival and disease-free survival.

Variable	Overall survival				Disease-free survival			
	Univariate analysis		Multivariate analysis		Univariate analysis		Multivariate analysis	
	HR (95% CI)	*p* value	HR (95% CI)	*p* value	HR (95% CI)	*p* value	HR (95% CI)	*p* value
Age (years), ≥ 65	0.682 (0.218, 2.139)	0.512	–	–	1.152 (0.661, 2.009)	0.618	–	–
Gender, female	0.524 (0.151, 1.820)	0.309	–	–	1.101 (0.502, 2.414)	0.810	–	–
Comorbidities, presence	1.696 (0.667, 4.316)	0.267	–	–	0.861 (0.526, 1.410)	0.553	–	–
Etiology, others	0.682 (0.218, 2.139)	0.512	–	–	0.923 (0.654, 1.302)	0.646	–	–
Cirrhosis, absence	0.404 (0.508, 3.875)	0.513	–	–	1.424 (0.678, 2.990)	0.351	–	–
Tumor size (cm), 2–3	1.053 (0.407, 2.723)	0.915	–	–	1.404 (0.852, 2.314)	0.183	1.794 (1.015, 3.169)	0.044
Abutting vessel, HV	0.647 (0.248, 1.686)	0.373	–	–	1.133 (0.697, 1.843)	0.615	–	–
AFP (ng/mL), > 20	0.267 (0.892, 5.759)	0.085	1.697 (1.226, 5.461)	0.019	0.382 (0.046, 1.578)	0.374	–	–
ALB (g/L), ≥ 35	0.659 (0.209, 2.077)	0.477	–	–	0.313 (0.060, 2.077)	0.167	–	–
TBIL (μmol/L), ≥ 17.1	2.829 (1.119, 7.148)	0.028	–	–	1.387 (0.811, 2.372)	0.232	–	–
ALT (U/L), ≥ 40	0.748 (0.266, 2.104)	0.582	–	–	2.155 (0.477, 9.417)	0.539	–	–
AST (U/L), ≥ 40	1.113 (0.218, 2.139)	0.830	–	–	1.151 (0.223, 5.397)	0.867	–	–
CTP grade, B	3.136 (0.406, 24.26)	0.273	–	–	3.136 (0.406, 24.26)	0.273	–	–
ALBI grade, 2–3	3.600 (1.178, 11.01)	0.025	2.071 (1.891, 5.410)	0.020	0.412 (0.080, 2.128)	0.290	–	–
Treatment modality, RFA	1.295 (0.507, 3.311)	0.589	–	–	0.940 (0.467, 1.890)	0.862	–	–

A Cox proportional hazards regression model for overall survival and disease-free survival was used. All variables were included in a multivariate stepwise Cox regression analysis. Only the variables with a *p* < 0.05 in the final model were presented. *HR*, hazard ratio; *CI*, confidence intervals; *OS*, overall survival; *DFS*, disease-free survival; *HBV*, hepatitis B virus; *HCV*, hepatitis C virus; *CTP*, Child-Turcotte-Pugh; *ALBI*, albumin-bilirubin ; *AFP*:α-fetoprotein; *ALB*: albumin; *TBIL*: total bilirubin; *AST*, aspartate aminotransferase; *ALT*, alanine aminotransferase; *PV*, portal vein; *HV*, hepatic vein; *RFA*, radiofrequency ablation

### Analysis of risk factors for LTP

**Fig. 3:**
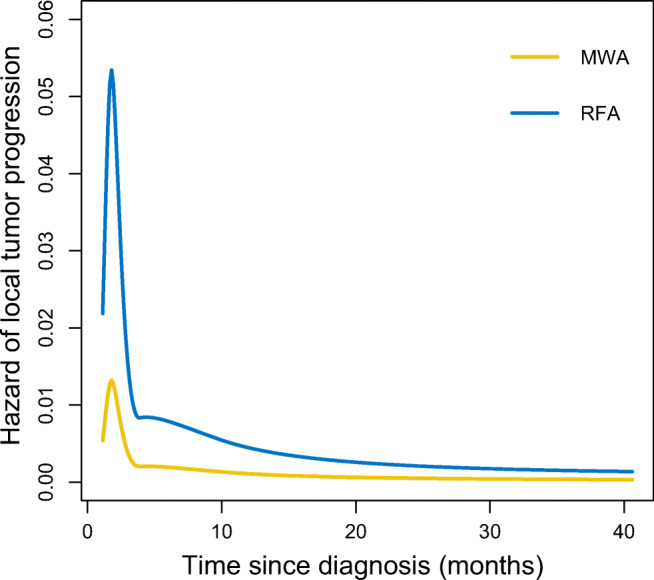
The estimated hazard of local tumor progression over time between the two treatment modalities. The hazard over time was estimated using the Royston Parmar model that uses a natural spline for the transformed baseline for log (time) with a log-log link

The results of univariate and multivariate step Cox regression analyses for LTP are summarized in Table 4. It was revealed that cirrhosis (HR: 0.284; 95% CI: 0.092–0.859; *p* = 0.028) and treatment modalities (HR: 6.826; 95% CI: 1.393–28.365; *p* = 0.017) had a significant effect on LTP. Furthermore, gender (HR: 0.148; 95% CI: 0.034–0.652; *p* = 0.012), cirrhosis (HR: 0.248; 95% CI: 0.074–0.836; *p* = 0.025), and treatment modalities (HR: 7.861; 95% CI: 1.642–37.635; *p* = 0.010) were found to be independent prognostic factors for LTP. Flexible parametric models indicated that the HR of LTP in RFA was elevated compared to that in the MWA group, over time until half a year after the initial procedure, with the hazard keeping steady after that (Fig. 3).

**Table 4:** Prognostic factor analysis for local tumor progression

Variable	Univariate analysis		Multivariate analysis	
	HR (95% CI)	*p* value	HR (95% CI)	*p* value
Age (years), ≥ 65	1.581 (0.487, 5.391)	0.446	–	–
Gender, female	0.384(0.104, 1.424)	0.151	0.148 (0.034,0.652)	0.012
Comorbidities, presence	0.773 (0.252,2.961)	0.651	–	–
Etiology, others	0.773 (0.252,2.961)	0.651	–	–
Cirrhosis, absence	0.284 (0.092, 0.859)	0.028	0.248 (0.074,0.836)	0.025
Tumor size (cm), 2–3	1.175 (0.384,3.593)	0.777	–	–
Abutting vessel, HV	1.313(0.439, 3.962)	0.626	–	–
AFP (ng/mL), > 200	0.554 (0.123, 2.508)	0.443	–	–
CTP grade, B	6.188 (0.798, 42.434)	0.081	–	–
ALBI grade, 2–3	0.471 (0.145,1.530)	0.210	–	–
Treatment modality, RFA	6.826 (1.393,28.365)	0.017	7.861 (1.642, 37.635)	0.010

A Cox proportional hazards regression model for overall survival and disease-free survival was used. All variables were included in a multivariate stepwise Cox regression analysis. Only the variables with a *p* < 0.05 in the final model were presented. *HR*, hazard ratio; *CI*, confidence intervals; *OS*, overall survival; *DFS*, disease-free survival; *HBV*, hepatitis B virus; *HCV*, hepatitis C virus; *CTP*, Child-Turcotte-Pugh; *ALBI*, albumin-bilirubin; *AFP*: α-fetoprotein; *ALB*: albumin; *TBIL*: total bilirubin; *AST*, aspartate aminotransferase; *ALT*, alanine aminotransferase; *PV*, portal vein; *HV*, hepatic vein; *RFA*, radiofrequency ablation

### Subgroup analysis for type of peritumoral vessel

There were no significant differences between the two ablation therapies with regard to OS and DFS in both periportal HCC and perivenous HCC (all *p* > 0.05, Table 5). However, we observed a significant interaction effect between ablation modality and type of peritumoral vessel on LTP (*p* for interaction: 0.034, Table 5). The LTP rate was significantly higher in the RFA group than in the MWA group for patients with periportal HCC (*p* = 0.045). However, this was not the case in patients with perivenous HCC (*p* = 0.116).

**Table 5:** Subgroup analysis according to the abutting vessel type

Endpoint	Abutting vessel type	HR (95% CI)	*p* value	*p* value for interaction
OS	Periportal HCCs (*N* = 80)	0.787 (0.108–5.751)	0.814	0.930
	Perivenous HCCs (*N* = 64)	1.216 (0.309–4.779)	0.780	
DFS	Periportal HCC (*N* = 80)	0.943 (0.458–1.941)	0.874	0.879
	Perivenous HCCs (*N* = 64)	1.041 (0.509–2.129)	0.912	
LTP	Periportal HCC (*N* = 80)	6.443 (1.046–39.69)	0.045	0.034
	Perivenous HCCs (*N* = 64)	5.733 (0.650–50.59)	0.116	

Interaction between the type of peritumoral hepatic vessel and treatment modalities was analyzed. *OS*, overall survival; *DFS*, disease-free survival; *LTP*, local tumor progression; *HCC*, hepatocellular carcinoma; *HR*, hazard ratio; *CI*, confidence intervals

## Discussion

To the best of our knowledge, our study represents the first study to compare the therapeutic outcomes of RFA and MWA as first-line therapies in patients with small single perivascular HCC. We found that OS and DFS outcomes between MWA and RFA in patients with small perivascular HCC were comparable. However, better local tumor control was achieved for the MWA group. MWA was an independent prognostic factor for LTP in perivascular HCC patients. It was associated with a shorter procedural duration and exhibited a lower ablative power compared to RFA. Therefore, MWA provides better curative effects and is more efficient for small perivascular HCC.

In this study, LTP risk was significantly higher in the first 6 months after the first-line therapy in the RFA group compared to the MWA group. Generally speaking, early recurrence (< 2 years) is usually characterized as intrahepatic metastasis resulting from incomplete ablation or tumor aggressiveness. In comparison, late recurrence (≥ 2 years) is attributed to staged progression or residual carcinoma in the situ in a liver with cirrhosis [19–21]. Based on this evidence, high early LTP rates observed after RFA first-line treatment could be explained by the following reasons. Firstly, some microsatellite nodules originating from perivascular HCC could not be detected in pre-treatment imaging. As the temperature increased slowly, part of the heat was removed by blood flow, which interfered with the elimination of cold areas in tissues containing microsatellite nodules adjacent to the major vessels. Secondly, rapid heating and a higher intratumoral temperature associated with MWA may restrict blood supply to tumor-bearing portal tributaries, thereby reducing the heat sink effect. Thirdly, incomplete ablation that is not detected on immediate CT or MRI may occur and contribute to regrowth of residual tumor following RFA, and this could manifest as early LTP during follow-up.

While the technical characteristics of RFA and MWA are quite similar, they exhibit several differences in their physical mechanisms of thermogenesis [22–24]. The significant difference is that during RFA, heat is confined to zones of high current density, while during MWA, it is generated in a fixed space around the antenna applicator. The ablation of nodules abutting the major vasculature is particularly challenging. To safely and effectively complete this task, exploring the heat sink effect in thermodynamic and electrical perspectives is required. Blood flow causes differences in convection and temperature as it transports heat from the tissue, which leads to the occurrence of an incomplete thermal field range. The heat sink effect is challenging when the ablative region is restricted to perivascular tissue during ablation therapy [25, 26]. Under these circumstances, low energy density within the ablative zone could not reach thermally toxic temperatures in nodules adjacent to the cooling vasculature. Therefore, RFA is not recommended for perivascular tumors because the synergy of electrical and heating sinks significantly increases the risk of insufficient ablation and local recurrence. In contrast, MWA is unconstrained by tissue conductance and quick heating and is, therefore, rarely affected by the defense of surrounding tissues. As a result, the heat sink effect has less influence on MWA treatment [27]. Bhardwaj et al histologically compared RFA, MWA, and cryoablation and found no perivascular hepatocyte survival in MWA. Perivascular hepatocyte survival was, however, observed within the ablated volume for cryoablation and RFA [28]. Moreover, Lee S et al reported that the prognosis of RFA for single, small perivascular HCC (diameter < 3 cm) [29], as a first-line treatment, was comparable to those of non-perivascular HCC. Based on these findings, we examined a similar study cohort comprising of patients with a perivascular HCC with a diameter of < 3 cm.

Based on the type of vessels adjacent to the tumor, perivascular HCCs are frequently classified into periportal HCC and perivenous HCC groups. In our study, subgroup analysis for the type of peritumoral vessels indicated that LTP for periportal HCC in the RFA group was significantly higher than that in the MWA group. However, LTP differences in perivenous HCC between the two ablation groups were not significant. These results indicate a higher risk of LTP in periportal than perivenous HCC after RFA, which may further affect survival outcomes. Our findings suggest that both RFA and MWA can be used as primary treatment options for perivenous HCC, whereas MWA is preferable for periportal HCC.

Although ablation therapy is effective in patients with HCC, the selection of ablation modalities should be evidence-based rather than clinician’s experience. However, few randomized controlled trials (RCTs) have recommended the optimal ablative option for patients with small perivascular HCCs. Our findings provide useful information for interventional radiologists in selecting the optimal ablative options for small perivascular HCC. In this study, we only observed two biliary complications after ablation. Both the RFA group and MWA group had one patient suffering from Biloma after ablation. Several reasons might account for the low incidence of biliary complications: (i) the study population had relatively small tumor diameter (< 3 cm); (ii) physicians pay great attention to perivascular HCC in ablation procedures; and (iii) physicians apply various methods to reduce complications, such as percutaneous transhepatic cholangial drainage with intraductal chilled saline perfusion.

Our study had several limitations. Firstly, potential selection and indication bias are inevitable due to the retrospective nature of the study design. Secondly, IPTW and multivariate analyses were used to enhance intergroup comparisons, and unidentified biases may have been beneficial for the MWA group. Thirdly, application of assistive techniques, including 3D VAPS, multi-modal image fusion guidance (MIFG), and artificial ascites or pleural effusion, is essential in choosing ablation modalities. These variables were not analyzed in this study due to the small sample size. Lastly, the different guidance methods used, including CT and US from multiple medical centers, may have led to artificial discrepancies in ablation techniques.

In conclusion, MWA provides better local tumor control over RFA as a first-line therapeutic option for small single periportal HCC. Interventional radiologists should evaluate the type of vessels adjacent to the tumor to balance the risk-benefit of ablation treatment in a single, small HCC.

## Supplementary Information

ESM 1 (DOCX 1675 kb)

## Abbreviations

AFP: Alpha-fetoprotein
ALB: Albumin
ALBI: Albumin-bilirubin
ALT: Alanine aminotransferase
AST: Aspartate aminotransferase
CTP: Child-Turcotte-Pugh
DFS: Disease-free survival
HCC: Hepatocellular carcinoma
IPTW: Inverse probability of treatment weighting
LTP: Local tumor progression
MWA: Microwave ablation
OS: Overall survival
PET: Positron emission tomography
RFA: Radiofrequency ablation
SMD: Standard mean difference
SR: Surgical resection
TBIL: Total bilirubin
