# Supplementary material for: Small single perivascular hepatocellular carcinoma: comparisons of radiofrequency ablation and microwave ablation by using propensity score analysis
Source: Eur Radiol. 2021 Jan 5;31(7):4764–73. doi: 10.1007/s00330-020-07571-5 (PMC8213545; doi:10.1007/s00330-020-07571-5)
Supplement: Supplementary file 1 — (DOCX 1675 kb) [file 330_2020_7571_MOESM1_ESM.docx]

**Supplementary Materials**

1. **Supplementary Methods**

**MWA Equipment**

The microwave unit (KY-2000; Kangyou Medical) was capable of producing 100 W of power at 2,450 MHz. The cooling tip needle antenna had a diameter of 1.9 mm (15 gauge) and a length of 18 cm.

**RFA Equipment**

We used commercially available electrode systems with generators (Cool-tip RF System [Covidien]) and an internally cooled, 17-gauge, 15 cm, electrode, with a 2 or 3 cm long ‎exposed ‎metallic tip.

**CT-guided Ablation Procedure**

The procedure of CT-guided radiofrequency ablation (RFA) is the same as that of microwave ablation (MWA). The RFA procedures were performed using a 16 multidetector-row CT (MDCT) scanner (Brilliance CT BigBore; Phillip Medical Systems). Ablation procedures were performed under the guidance of one of three radiologists, J.H.H., Z.M.H, and C.A. with 25, 8 and 5 years of experience, respectively. During the exercise, we used commercially available Cool-tip RF electrode systems. Before ablation, we administered local anaesthesia comprising 1% lidocaine (Yi you) subcutaneously while analgesia was intravenously administered using 2-3ng/ml remifentanil to moderately sedate each patient. We performed biopsy under CT-guided, before RFA, using an automatic biopsy gun bearing an 18-gauge cutting needle. The electrode was then repeatedly inserted with slight changes to the angle of the tip until the ablation zone covered the entire tumor area with a margin of safety > 0.5 cm. When the tumor is adjacent to the hilar area or the main bile duct, we must be cautious. When the ablation process, CT images show biliary gas, we must reduce the ablation power and time to avoid bile duct damage.

**US-guided Ablation Procedure**

The procedure of US-guided radiofrequency ablation (RFA) is the same as that of microwave ablation (MWA). The MWA procedures were performed under the guidance of ultrasound (US) ‎by three interventional radiologists (P.L., X.L.Y., and J.Y., with 20, 20 and 10 years of ‎experience in MWA, respectively). For this, a KY2000 MWA system (Kangyou Medical Instruments) comprising two ‎autonomous MW generators, two flexible coaxial cables and two water-pumping ‎machines were used. Patients were first administered with intravenous anaesthesia containing 6-12 mg/kg/h propofol and 1-2 mg/kg ketamine during the ablation procedure in the operating room. ‎The tumours and tumour-feeding arteries were not well visualized under the conventional ‎US, necessitating a contrast-enhanced ultrasound (CEUS)-guided ablation. An automatic biopsy gun, with an 18 gauge‎ ‎cutting needle, was used to carry out a US-guided biopsy before ‎ablation. Consequently, a 15 gauge‎ antenna was directly and percutaneously inserted into the tumor. Multisite ablation was performed according to the preoperative ‎plan, with MW power range controlled between 50 - 60 W, and ablation duration refined to 5-10 minutes. The antenna ‎was then repeatedly inserted until the ablation zone was achieved according to the ‎treatment plan. ‎CEUS was performed with SonoVue (Bracco, Milan, Italy) to ‎accurately and preoperatively target the tumour and assess the postoperative ablation effect immediately ‎after ablation.‎ We used percutaneous transhepatic cholangial drainage with intraductal chilled saline perfusion (PTCD-ICSP) to avoid the bile duct damage. For the biliary tube drainage, we used a 6 or 8.5F drainage catheter (COOK) with more side-holes. The tip of the tube was positioned in the hepatic duct close to the lesion and injected an ultrasound contrast agent (Sonovue BR1; Bracco SpA) into the tube before ablation. Frozen saline was thawed just before ablation, and was perfused during the whole ablation procedure through the tube by drip infusion with the speed of 0.2 ml/s, then the speed was changed to 0.05 ml/s until two hours after ablation.

1. **Supplementary Figures**

**sFigure 1.** A three-dimensional image generated by the three-dimensional visualization ablation planning system for demonstrating the spatial relationship between tumours and major vessels.

**sFigure 2.** A follow-up medical record example of radiofrequency ablation (RFA) for perivascular tumours.

**sFigure 3.** A follow-up medical record example of microwave ablation (MWA) for perivascular tumours.


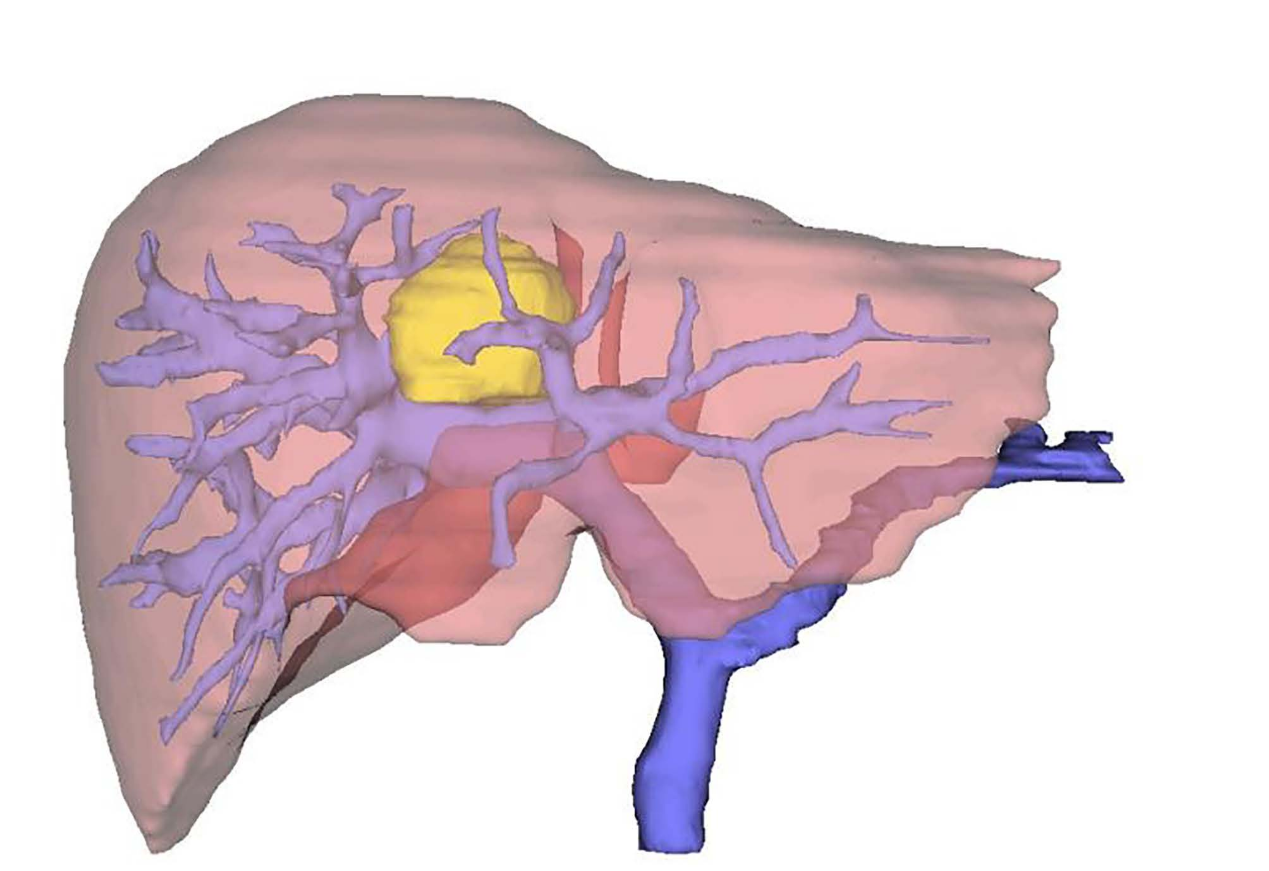


**sFigure 1.** A three-dimensional image generated by the three-dimensional visualization ablation planning system for demonstrating the spatial relationship between tumours and major vessels.

**
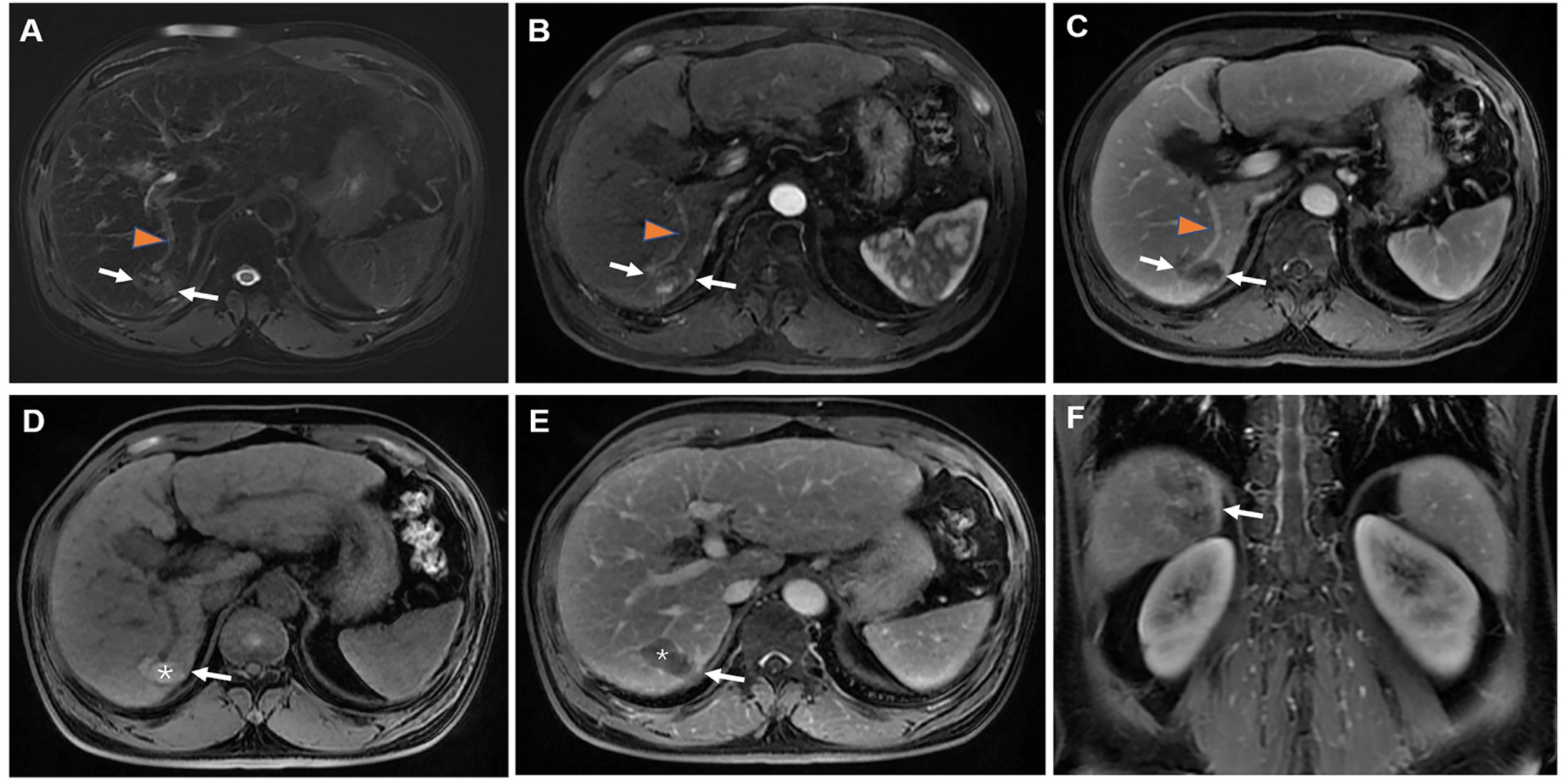
**

**sFigure 2.** A follow-up medical record example of radiofrequency ablation (RFA) for perivascular tumours.

**
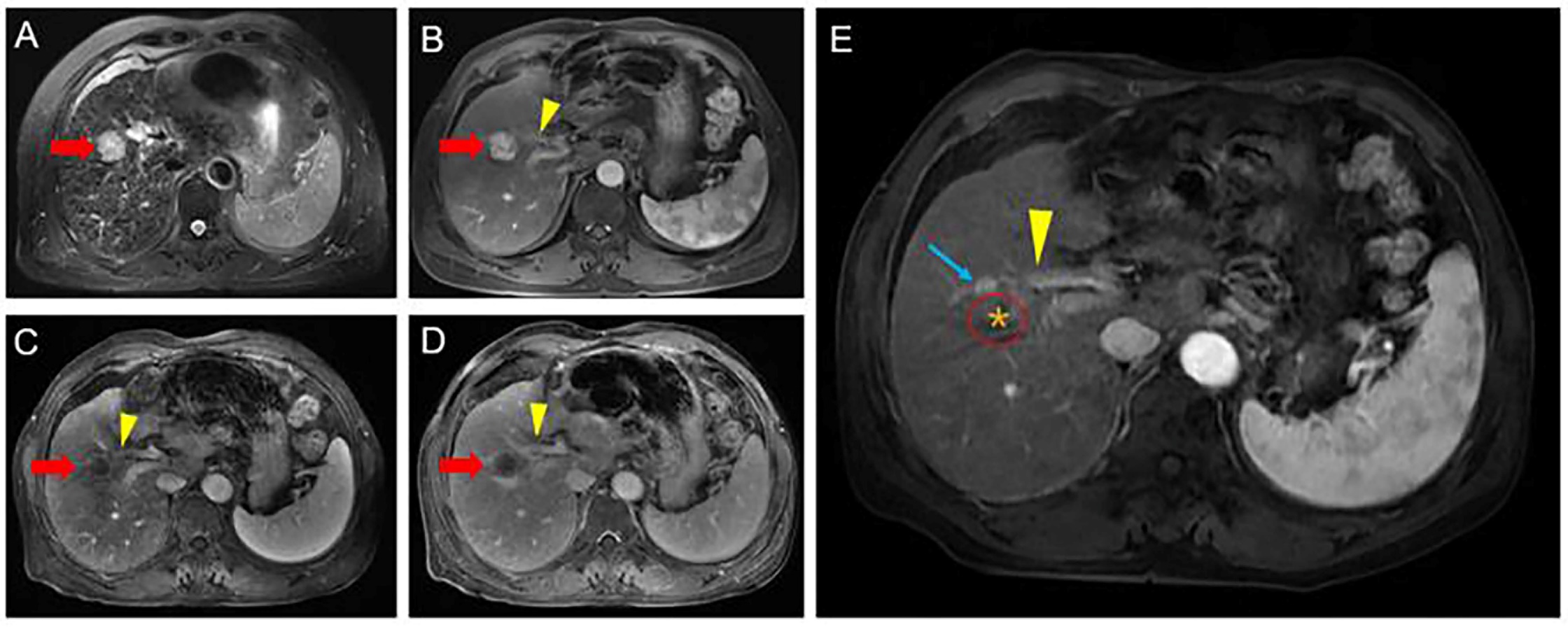
**

**sFigure 3.** A follow-up medical record example of microwave ablation (MWA) for perivascular tumours.
